# Correspondence Between Student and Teacher Reports of School Climate: Ideas for Strengthening School Behavioral Health Programming

**DOI:** 10.3390/bs16060859

**Published:** 2026-05-27

**Authors:** Alena Quinn, Halle R. Singer, Abigail Westbrook, Samuel D. McQuillin, Brooke E. Chehoski, Mark D. Weist

**Affiliations:** Department of Psychology, University of South Carolina, Columbia, SC 29208, USA

**Keywords:** school behavioral health, school climate, school safety, teachers, students, implementation support

## Abstract

Comprehensive school behavioral health (SBH) programs offer an integrated approach for mental health support. However, program success hinges on critical school-level factors, particularly school climate. While prior research has examined the discrepancy between student and teacher perceptions of school climate, there is uncertainty surrounding how correspondence of school climate perceptions may inform SBH implementation and continuous improvement. This exploratory study investigates student and teacher perceptions of school climate across 19 schools implementing a comprehensive SBH program within a large-scale randomized controlled trial. School climate data were collected during spring 2022 using the U.S. Department of Education School Climate Survey, which assesses school climate domains of engagement, safety, and environment. Pearson correlations and Cohen’s *d* were conducted using aggregate school-level student and teacher reports to examine correlation and mean-level differences across domains. Results revealed significant, moderate to high correspondence between student and teacher perceptions in domains of safety and environment, particularly across subdomains of physical safety, bullying/cyberbullying, substance abuse, mental health, and discipline. Findings indicate how correspondence in areas of school climate, such as safety and environment, may inform the implementation and improvement of school-wide SBH programming.

## 1. Introduction

School behavioral health (SBH) programs have gained momentum as a framework for delivering coordinated, evidence-based support within school settings (Weist et al., 2020). Extensive research has indicated that the implementation of SBH can lead to positive student outcomes such as improved social, emotional, and behavioral functioning (Weist et al., 2022). Despite its promise, the successful implementation and sustainability of SBH programs are influenced by key school-level conditions, particularly school climate (Splett et al., 2023). A growing body of research suggests that school climate plays a foundational role in supporting effective mental health service delivery and implementation (Lyon & Bruns, 2019; Splett et al., 2023), yet less is known about how the perceptions of students and teachers can inform SBH implementation and improvement efforts when these perceptions correspond. Thus, this exploratory study examines the extent to which students and teachers may correspond in their perceptions of school climate and how this correspondence may guide school-level SBH implementation and improvement efforts.

### 1.1. School Behavioral Health Programs

Embedding mental health supports within school settings reduces barriers to care and fosters environments where mental health and help-seeking behaviors are normalized (Aguirre Velasco et al., 2020; Lindow et al., 2020). As a result, students, particularly those from underserved populations, are significantly more likely to initiate and complete mental health services when they are provided in schools compared to community settings (Ali et al., 2019; Jaycox et al., 2010). Despite these advantages, SBH systems continue to encounter persistent implementation challenges that limit access to quality service delivery (Brown & Carrington, 2025; Gronholm et al., 2018). The Interconnected Systems Framework (ISF) aims to address these challenges by promoting structured collaboration between schools and community mental health systems (Barrett et al., 2025; Eber et al., 2019; Weist et al., 2020). When implemented effectively, integrated SBH programs have been shown to improve student outcomes such as engagement, attendance, and graduation rates, while reducing behavioral incidents and the need for restrictive placements (Flannery et al., 2014; Kern & Rusnak, 2024; Taylor et al., 2017; Weist et al., 2022). These improvements can contribute to a more positive and predictable school climate, characterized by stronger relationships and an enhanced sense of safety and belonging among students and staff (Thapa et al., 2013). Beyond student outcomes, a positive school climate may also facilitate effective SBH implementation. Positive school climates have been associated with increased staff capacity, self-efficacy, and job satisfaction (Brabson et al., 2019). In this way, school climate may serve as a necessary facilitator to enhance implementation support by increasing student and staff willingness and engagement in SBH services.

### 1.2. School-Level Barriers to SBH Implementation

While the ISF provides a clear framework for integrating SBH services across multiple tiers, school systems were not originally designed to support the integrated, interdisciplinary approach on which SBH and ISF depend (Atkins et al., 2010; Eber et al., 2019). As a result, school systems have to navigate limited organizational capacity, leadership constraints, and competing priorities (Mullen & Gutierrez, 2016; Williams & Beidas, 2018). These school-level limitations particularly impact effective Tier 1 (universal) and Tier 2 (targeted) implementation, which require sustained leadership and engagement. For example, Tier 2 supports may be disrupted by unclear referral processes, limited time for team-based meetings, and siloed or fragmented communication between staff (Lawson et al., 2022; Mellin et al., 2010), emphasizing the need for coordinated leadership. Also, the effectiveness of Tier 2 interventions depends substantially on student willingness to participate in the interventions (Lyon & Bruns, 2019; Weist et al., 2017; Zabek et al., 2023), reflecting efforts towards increased engagement. Across both tiers, school-level organizational readiness plays a critical role in determining implementation success (Lyon & Bruns, 2019). Schools vary widely in their capacity to adopt new initiatives, align with existing systems, and sustain the practice over time. The importance of understanding school-level barriers has become increasingly clear (Greenham et al., 2019; Thapa et al., 2013), as these interconnected factors collectively shape overall school climate and the conditions under which SBH implementation can succeed.

### 1.3. Leveraging School Climate to Advance SBH Implementation

School climate refers to the overall atmosphere within a school, including the physical environment, social relationships, and organizational norms that influence how students and staff feel, interact, and function (National School Climate Center, 2021; Thapa et al., 2013). Research evidence underscores the importance of a positive school climate with significant associations between climate dimensions (e.g., relationships, perceptions of safety, sense of connectedness, physical environment, etc.) and student outcomes, including academic success, social–emotional well-being, and behavior (Leurent et al., 2021; Moore et al., 2023). Complementary findings from other studies show that students in schools with more positive climates report fewer mental health concerns, suggesting that school climate can play an integral role in advancing SBH effectiveness (Franco et al., 2022; Podiya et al., 2025). These associations make clear that fostering a healthy school climate is not an ancillary concern, but a necessary school-level condition for effective SBH implementation.

As a key domain of school climate, school safety affects effective SBH implementation as it is collectively experienced by both students and staff. Shared positive perceptions of safety can increase time for school staff to devote attention, resources, and capacity towards SBH programming, while also enabling students to engage in services without fear or distraction (Aldridge & McChesney, 2018; Franco et al., 2022; Thapa et al., 2013). Exposure to violence, bullying, and perceived physical and emotional threats can influence both students’ and teachers’ sense of safety and belonging within the school (Franco et al., 2022; Moore et al., 2023). These conditions are foundational to student engagement in SBH services (DePaoli & McCombs, 2023; Fenizia & Parrello, 2025). Schools that experience increased safety concerns often fall short of time and resources to implement and promote SBH efforts (Robinson et al., 2025). Additionally, heightened safety measures and discipline experiences may contribute to a mistrust between students and adults, decreasing their willingness to seek support (Del Toro & Wang, 2021; Gottfredson et al., 2020). Accordingly, prioritizing school safety may foster the interpersonal and organizational conditions necessary for effective SBH implementation (Glisson, 2015; Thapa et al., 2013).

To help schools address these multifaceted components of climate, the U.S. Department of Education has outlined a three-factor model consisting of engagement, safety, and environment (National Center for Education Statistics, n.d.). These domains offer a structured approach to examining key components of school climate and how school climate can influence both learning and mental health (National Center for Education Statistics, 2015). When used alongside SBH efforts, each domain can serve as an entry point for identifying school-wide strengths and gaps that affect student outcomes. For instance, a school may demonstrate high levels of engagement but low safety, signaling a need to implement strategies that promote respectful behavior and language to make students and school staff feel more secure at school. When schools are made aware of their school climate concerns, they can target areas that support effective SBH implementation.

Existing research has focused on the impact of school climate on students and their discrepancies in perceptions from teachers (MacGregor et al., 2024; Wang & Degol, 2016). A critical but less explored avenue is student and teacher perceptions of school climate that correspond, as they may experience SBH efforts differently or share patterns of strengths and challenges related to school climate. Correspondence is defined as the degree of shared perceptions across students and teachers at the school level. Accordingly, strong correspondence across school climate domains between students and teachers may suggest a shared understanding and readiness for coordinated action, signaling that implementation is likely to be more feasible (Konold et al., 2018; Mitchell et al., 2010). This can function as valuable guidance for SBH teams using domains of school climate to inform school-level action. Conversely, discrepancies across domains may reveal areas requiring targeted engagement, communication, and professional development before intervention can be effectively implemented (Abshier et al., 2023). Examining correspondence extends prior research by shifting the focus to examining shared perceptions within the school context rather than only where perceptions may differ. For SBH teams, climate may operate as a decision-making lens that helps identify both opportunities and barriers to implementing SBH programs. When student and teacher perspectives correspond, schools are better able to identify areas of needed improvement and tailor SBH programming to those unique contexts. For example, shared student and teacher concerns about emotional safety may indicate a need for Tier 1 supports, such as school-wide social–emotional learning, trauma-informed staff training, mental health literacy, and programs that promote well-being among students and staff (Figas et al., 2024; Yanek et al., 2022).

### 1.4. Present Study

The present exploratory study is a secondary analysis of school climate data collected as part of a larger randomized, controlled trial examining comprehensive SBH models (Weist et al., 2026). While the original study investigated intervention-related outcomes, this current study explored corresponding perceptions between students and teachers to identify areas of school climate that may facilitate targeted SBH implementation and improvement efforts, within and across 19 participating middle schools. The current study aims to address the following questions: (1) To what extent do student and teacher perceptions of school climate correspond across domains of engagement, safety, and environment? (2) Which domains and/or subdomains demonstrate the strongest correspondence between students and teachers? To address these questions, Pearson’s correlation analysis was used to analyze the correspondence between student- and teacher-reported school climate domains and subdomains to understand critical aspects of school climate that may contribute to the improvement of SBH implementation. Additionally, Cohen’s *d* was calculated to analyze mean-level differences between student and teacher perceptions of school climate. Because the purpose of this study was to examine shared perceptions of school climate, intervention analysis was not included. We hypothesized that students’ and teachers’ perceptions of school climate domains that positively correlate, particularly within the school safety domain, will suggest correspondence. Stronger correlations of specific school climate domains may indicate having a shared understanding amongst students and teachers, which may lead to actionable areas to support SBH implementation.

## 2. Methods

### 2.1. Participants

The methodological detail of the original study that served as the basis of this research is well documented in the original study (Weist et al., 2026), with the most relevant information discussed here. The original study involved 20 schools in the United States from one mid-Atlantic state and one Southern state, participating in a larger randomized controlled trial examining comprehensive SBH effectiveness. Schools were selected in collaboration with district and community partners to ensure representation across diverse settings. Participating schools represented large student populations across rural, suburban, and urban communities in both states. For this exploratory study, one of the schools did not provide sufficient school climate data and, therefore, was excluded from further analysis. Student participants included enrolled students present during survey administration in spring 2022 and whose parents/caregivers did not decline their child’s participation. Teacher participants included instructional staff employed at participating schools during spring 2022.

Of the 19 schools included, there were 1188 students across both states (614 male, 493 female, 40 non-binary/gender nonconforming, 29 “other,” and 12 prefer not to answer). Additionally, there were 303 teachers across both states (263 male, 36 female, 1 non-binary/gender nonconforming, and 3 “other”). Students’ age range and additional demographics (including socioeconomic status, race, and ethnicity) are available in the original study with accompanying tables (Weist et al., 2026).

### 2.2. Procedures

The present exploratory study utilized cross-sectional school climate data collected during the spring 2022 semester. Although schools were originally part of different randomized conditions of coordinated SBH programming, intervention conditions were not included in the present study. The current study examines correspondence between student and teacher perceptions, rather than estimating intervention effects. Additionally, all participating schools were engaged in SBH implementation to varying degrees, and school climate data were collected as a shared measurement across conditions, making condition-specific comparisons beyond the scope of these analyses.

#### School Climate

The School Climate Survey was administered to students and teachers across participating schools during a designated one-week administration window in spring 2022. Although data were collected across multiple years in the original study, the present analysis was restricted to the spring 2022 semester, as it provided the most complete survey responses for both students and teachers, making direct comparisons between groups available. Sampling procedures differed slightly by state due to differences in school size and available resources. In the Southern state schools, one core subject class per grade level was randomly selected to participate in the survey to obtain a representative sample. In the mid-Atlantic state schools, which were smaller in enrollment, all core classes in grades six through eight were invited to participate. Caregivers received an opt-out consent letter prior to administration with instructions to return it if they did not authorize their student to participate. Teachers who administered the survey were provided instructions, a script, and the survey link for the students to complete it digitally. Surveys were completed digitally in the Southern state schools and by paper format in the mid-Atlantic state schools due to limited technology access. Trained research assistants entered the data electronically, with review from senior research staff to reduce the possibility of error upon data entry.

### 2.3. Measurements

The current study focused specifically on two measures, student-reported and teacher-reported school climate surveys, to examine the correspondence between these two groups.

#### School Climate Survey

The U.S. Department of Education School Climate Survey (SCS) was administered to students, teachers, and non-instructional staff at all participating schools. The SCS measured various dimensions of school climate, with each item corresponding to a specific domain (i.e., engagement, environment, and safety) that also contains various subdomains. Student and teacher versions were designed as parallel instruments where items differed in wording and were conceptually mapped onto a specific domain and/or subdomain. The SCS has been psychometrically validated in large, nationally represented samples and demonstrates strong measurement properties, including internal consistency and construct validity that support the three-domain structure (Bradshaw et al., 2014; National Center for Education Statistics, 2015). Validation research reported acceptable to strong reliability for the Engagement (α = 0.88), Safety (α = 0.78), and Environment (α = 0.90) domains in a districtwide sample of middle and high schools (Ryberg et al., 2020). Scaled domain and subdomain scores for each school were calculated using item parameters based on a Rasch model. These aggregate scaled scores are categorized into three levels: least favorable (300 or below), favorable (300 to 400), and most favorable (400 to 500 and above).

The *Engagement* domain evaluated key aspects of school connectedness and has three subdomains: Cultural Linguistic Competence, Relationships, and School Participation. Cultural and Linguistic Competence refers to the ability to effectively interact with people from diverse cultural backgrounds and to communicate across languages in a respectful and inclusive manner. Relationships refer to the quality of interactions between students and staff, including feeling supported. School Participation refers to the opportunities students have to engage in classroom activities and take part in extracurricular programs. Examples of items in this domain include “Adults working at this school treat all students respectfully,” for students, and “This school emphasizes showing respect for all students’ cultural beliefs and practices”, for teachers.

The *Safety* domain measured how safe and supported students feel at school, including their ability to focus on learning and their sense of belonging, and has four subdomains: Emotional Safety, Physical Safety, Bullying/Cyberbullying, and Substance Abuse. Emotional Safety refers to students feeling understood, respected, and happy to be at school. Physical Safety measures the students’ feelings of safety in their school environment, as well as in spaces related to school. Bullying/Cyberbullying is the extent to which a student experiences harmful repeated behavior, whether in person or online. Substance abuse refers to the use or attempted use of alcohol or drugs by students while at school or at school-related events. Examples of items in this domain include “I feel safe at this school” and “I feel like I belong.”

The *Environment* domain assessed well-maintained and welcoming facilities, clear expectations, a focus on productivity and inclusion, and has five subdomains: Physical Environment, Instructional Environment, Physical Health, Mental Health, and Discipline. Physical Environment refers to the cleanliness, comfort, and overall condition of the school’s facilities. Instructional Environment refers to the support and encouragement students receive from teachers. The Physical Health subdomain was only evaluated by teachers to address the school’s emphasis on promoting healthy eating and other health needs. Mental Health involves the students feeling cared for and supported by school staff, with the ability to discuss challenges. Discipline pertains to setting clear expectations for behavior and recognizing students for positive behavior. Examples of items in this domain include “the school grounds are kept clean” for students and “the school grounds look clean and pleasant” for teachers.

### 2.4. Data Analytics Plan

We analyzed correlations between each corresponding school climate domain and subdomain for student and teacher ratings across the 19 schools. Because the current analyses focused specifically on school-level correspondence between students and teachers, schools from both intervention conditions were combined into a single analytic sample. All analyses were conducted using RStudio (Version 2025.5.1.513, Posit Team, 2025). Pearson correlations between student-reported and teacher-reported school climate domains were conducted to understand the degree of correspondence or discrepancy amongst students and teachers within and across schools. To analyze mean-level differences, Cohen’s *d* was calculated to further interpret the relationship between student and teacher perceptions of school climate. Given that only school-level aggregate scores were available for analysis, Pearson correlations were selected as the most appropriate method for exploring the degree of linear association between aggregate student and teacher-reported perceptions of school climate. By understanding the correspondence between student and teacher-perceived school climate across domains, we tested our hypothesis that corresponding perceptions will positively correlate. Stronger alignment in specific domains may emphasize shared perceptions that may guide targeted SBH interventions and enhance implementation efforts. The findings of this exploratory study involved multiple correlations across school climate domains and subdomains, and associations that did not reach statistical significance should be interpreted cautiously.

## 3. Results

Table 1 and Table 2 include demographic data for all participants in this analysis. Table 3 includes descriptive data and Cohen’s *d* values for student and teacher School Climate Survey (SCS) reports during the spring 2022 semester. Table 4 includes Pearson correlation analyses between student and teacher reports of school climate. Pearson correlations and Cohen’s *d* were presented together to distinguish school-level correspondence from mean-level agreement. In this context, Pearson correlations indicate whether student and teacher perceptions varied similarly across schools, whereas Cohen’s *d* indicates whether students and teachers differed in the overall amount or level of their perceptions.

Results showed that several domains and subdomains demonstrated positive school-level correspondence, but this correspondence did not always indicate convergence in the level of concern reported by students and teachers. It was hypothesized that student and teacher perceptions of their school’s climate would positively correlate, indicating correspondence between student and teacher perceptions. Results indicated a range of significant, moderate (*r* ≥ 0.3) to strong (*r* ≥ 0.5) positive correlations between student and teacher ratings, suggesting correspondence in perceptions in some areas of school climate. However, some significant correlations had large mean-level differences, suggesting students and teachers ranked school climate similarly, but not necessarily as evidence that they perceived the same degree of concern.

### 3.1. Engagement Domain

Across the Engagement domain, students consistently reported more favorable perceptions of school engagement than teachers, with large, standardized mean-level differences across the domain. Students reported higher scores of overall Engagement (*M* = 290.80, *SD* = 14.49) than teachers (*M* = 257.95, *SD* = 20.84), *d* = 1.86. However, student and teacher reports of Engagement were not significantly correlated, *r* = 0.34, *p* = 0.16. This pattern suggests a large mean-level difference without clear school-level correspondence, indicating divergence rather than convergence in student and teacher perceptions of engagement. For Cultural and Linguistic Competence, students reported more favorable perceptions (*M* = 282.76, *SD* = 19.18) than teachers (*M* = 256.59, *SD* = 19.05), *d* = 1.37. Yet, student and teacher reports showed a moderate positive correlation that approached statistical significance, *r* = 0.45, *p* = 0.052. This may suggest a potential partial overlap in correspondence, as students and teachers did not report similar levels of perceived cultural and linguistic competence. For Relationships, students also reported higher scores (*M* = 296.55, *SD* = 15.74) than teachers (*M* = 247.36, *SD* = 17.71), *d* = 2.94. However, student and teacher reports were not significantly correlated, *r* = 0.28, *p* = 0.24. Finally, for Participation, students reported more favorable perceptions (*M* = 287.03, *SD* = 15.58) than teachers (*M* = 264.62, *SD* = 30.99), *d* = 0.96; however, there was no evidence that students and teachers corresponded with their perceptions of school participation (*r* = 0.02, *p* = 0.94). These findings suggest that students tended to rate school engagement more favorably than teachers, and evidence of correspondence between student and teacher perceptions varied and was generally not significant.

### 3.2. Safety Domain

For the Safety domain, students reported more favorable overall safety (*M* = 293.74, *SD* = 33.13) than teachers (*M* = 284.76, *SD* = 16.08), *d* = 0.37. Student and teacher reports of safety were significantly correlated, *r* = 0.75, *p* = 0.0002. This pattern of strong correspondence with small mean-level differences indicates convergence for how students and teachers perceive their school’s overall safety. For Physical Safety, students reported somewhat more favorable perceptions (*M* = 297.49, *SD* = 38.93) than teachers (*M* = 283.88, *SD* = 28.49), *d* = 0.40. Student and teacher reports of physical safety were found to be significantly correlated, *r* = 0.58, *p* = 0.009. Similar to the overall Safety domain, this pattern suggests meaningful convergence, such that students and teachers appeared to report physical safety similarly, with relatively small mean-level differences compared to other subdomains. For Bullying/Cyberbullying, students reported more favorable perceptions (*M* = 309.57, *SD* = 35.54) than teachers (*M* = 286.72, *SD* = 17.81), *d* = 0.86, and these were positively and significantly correlated, *r* = 0.69, *p* = 0.001. Although this indicates strong school-level correspondence, the large mean-level difference in student and teacher ratings suggests a potential partial overlap in correspondence rather than full convergence. For Substance Abuse, students reported substantially more favorable perceptions (*M* = 204.58, *SD* = 89.02) than teachers (*M* = 299.12, *SD* = 24.13), *d* = −1.67. Despite the large difference in mean levels, student and teacher reports were significantly positively correlated, *r* = 0.51, *p* = 0.02. Therefore, this finding suggests that although schools that rated substance abuse more favorably by students also tended to be rated more favorably by teachers, the large mean-level difference indicates only partial overlap in corresponding perceptions of related concerns. For the Emotional Safety subdomain, students reported more favorable perceptions (*M* = 310.84, *SD* = 22.15) than teachers (*M* = 283.94, *SD* = 21.77), *d* = 1.23. Student and teacher reports of emotional safety were not significantly correlated, *r* = 0.19, *p* = 0.43. This pattern may suggest divergence in perceptions of emotional safety.

### 3.3. Environment Domain

For the Environment domain, students reported more favorable perceptions of the school environment (*M* = 301.15, *SD* = 14.22) than teachers (*M* = 272.20, *SD* = 20.46), *d* = 1.67. Student and teacher reports were positively and significantly correlated, *r* = 0.48, *p* = 0.04, indicating moderate correspondence between student and teacher perceptions of the school environment. This pattern suggests that although students and teachers reported corresponding perceptions of overall environments, students reported substantially more favorable perceptions than teachers. For Mental Health, students reported substantially more favorable perceptions (*M* = 317.64, *SD* = 20.76) than teachers (*M* = 255.55, *SD* = 22.78), *d* = 2.85. Student and teacher reports were positively and significantly correlated, *r* = 0.60, *p* = 0.007, suggesting moderate-to-strong correspondence in perceptions of the school’s mental health environment. This combination of correspondence and large mean-level difference suggests that students and teachers tended to correspond on school-level mental health, but teachers reported much less favorable perceptions overall, indicating a potential partial overlap. For Discipline, students reported slightly more favorable perceptions (*M* = 284.43, *SD* = 13.09) than teachers (*M* = 276.69, *SD* = 24.98), *d* = 0.41. Student and teacher reports were positively and significantly correlated, *r* = 0.61, *p* = 0.005, indicating moderate-to-strong correspondence across schools. This pattern of correspondence with relatively small-to-moderate mean-level differences suggests greater convergence than in other Environment subdomains. For Physical Environment, students reported more favorable perceptions (*M* = 322.63, *SD* = 31.30) than teachers (*M* = 277.48, *SD* = 39.93), *d* = 1.27. However, student and teacher reports were not significantly correlated, *r* = 0.33, *p* = 0.17, suggesting limited evidence of correspondence. This pattern of large mean-level differences with limited evidence of correspondence may indicate divergence in perceptions of the physical school environment. For Instructional Environment, students reported less favorable perceptions (*M* = 281.55, *SD* = 12.54) than teachers (*M* = 288.34, *SD* = 13.50), *d*= −0.52. Student and teacher reports of instructional environment were not significantly correlated, *r* = 0.07, *p* = 0.79, indicating little evidence of school-level correspondence. The Physical Health subdomain was not included in the student-teacher correlation analyses because parallel student-report items are not administered on the SCS.

## 4. Discussion

The present exploratory study examined the correspondence between student and teacher perceptions of school climate across the Engagement, Safety, and Environment domains. Overall, student and teacher reports had the strongest correspondence in the Safety domain, followed by the Environment domain and select subdomains (e.g., Physical Safety, Bullying/Cyberbullying, Substance Abuse, Mental Health, and Discipline). In contrast, Engagement-related perceptions showed weaker and nonsignificant correspondence. These findings should be interpreted as exploratory evidence of association. As such, the results are best understood as preliminary findings that may help identify areas of school climate for further inquiry by SBH teams. Significant correlations suggest that school climate rated more favorably (i.e., less concern) by students also tended to be rated more favorably by teachers in certain domains. However, correlations do not necessarily indicate whether students and teachers reported the same overall level of concern. Therefore, correlations were reported alongside mean-level differences to interpret the degree to which student and teacher perceptions appear to correspond.

### 4.1. Student-Teacher Correspondence on School Climate

We hypothesized that student and teacher reports of school climate would positively correlate, indicating agreement in their perceptions of their school’s climate. Results reveal moderate to strong, positive correlations across the Safety and Environment domains, supporting the hypothesis in specific areas of school climate. The strongest pattern of correspondence emerged in the Safety domain. Student and teacher reports were significantly positively correlated for overall Safety, Physical Safety, Bullying/Cyberbullying, and Substance Abuse. These findings may suggest that, across schools, students and teachers evaluated safety-related school climate similarly, such that schools rated more favorably by students for overall Safety and Physical Safety, also tended to be rated more favorably by teachers. When compared to other domains, smaller mean-level differences for overall Safety and Physical Safety may further reflect greater convergence between students and teachers in these areas.

Additionally, several significant correlations had discrepancies in mean levels, suggesting partial alignment overlap across students and teachers. Regarding Substance Abuse, student and teacher reports were significantly positively correlated, suggesting that students and teachers showed similar patterns across schools. However, students on average reported more favorable (i.e., less concern) perceptions of substance abuse than teachers. This may be interpreted to mean that while students and teachers identified similar patterns of relatively greater or lesser substance abuse concerns, they differed in the overall level of concern reported. A similar pattern was found in the Environment domain. Student and teacher reports were significantly positively correlated for the overall Environment domain, as well as the subdomains of Mental Health and Discipline. Across these areas of school climate, students on average reported more favorable perceptions than teachers. In these areas, students and teachers corresponded in their perceptions of school environment, mental health, and disciplinary action. However, students and teachers differed in their average ratings, suggesting that although they reported concerns in a similar direction, they did not perceive the same degree of concern across these areas.

In contrast, student and teacher reported perceptions of the Engagement domain showed little evidence of correspondence. Student and teacher reports were not significantly correlated for the overall Engagement domain or the Relationships and School Participation subdomains. Although students generally reported more favorable perceptions than teachers across these areas, the nonsignificant correlations suggest that, although students on average reported more favorable perceptions of engagement, teachers on average reported more engagement-related concerns. Additionally, Cultural and Linguistic Competence showed a positive association that approached but did not reach statistical significance. Taken together, these findings suggest that Engagement may be an area of school climate where student and teacher perceptions are more distinct, reflecting that students and teachers may engage differently within the school context.

There were several other subdomains that showed limited evidence of correspondence despite mean-level differences. The subdomains of Emotional Safety, Physical Environment, and Instructional Environment were not significantly correlated between students and teachers. These findings may suggest that student and teacher perspectives in these areas may highlight different experiences or interpretations of engagement relating to school climate.

### 4.2. Leveraging School Climate to Support SBH Implementation

The combination of observed correspondence and mean-level differences across safety and environment highlights the importance of taking a nuanced perspective on interpreting and responding to universal school climate data. As such, these findings may have plausible implications for how schools use climate data to inform SBH implementation. Examining the correspondence as well as mean-level differences in student and teacher perceptions may provide further contextual information for SBH teams to identify areas for further inquiry and potential improvement. Shared perceptions of climate may indicate opportunities for school-wide efforts to sustain or increase strong school climate, as both students and teachers interpret these domains similarly. Considering areas of correspondence may enhance coherence in school implementation efforts by incorporating both student and teacher perspectives to inform school-level initiatives that, in turn, can support outcomes within SBH programs.

Areas showing significant positive correspondence may serve as useful starting points for SBH teams. When student and teacher perceptions are reported in a similar direction of relatively greater or lesser concern, it may suggest a shared experience or interpretation of relative strengths or areas for improvement. Identifying these patterns may support school-wide alignment of SBH services. Schools experiencing elevated safety concerns, such as violence, bullying, or substance use, often need to divert leadership attention and resources towards crisis response (Thapa et al., 2013; U.S. Department of Education, 2023). However, SBH teams assessing perceptions of safety may identify plausible implications for universal Tier 1 implementation, such as school-wide initiatives including positive behavioral supports, mental health literacy, and trauma-informed practices (Barrett et al., 2015; Kern & Rusnak, 2024). At the Tier 2 level, corresponding student and teacher perceptions of safety concerns may help teams identify targeted supports for students facing bullying, violence, or substance use (Thapa et al., 2013; U.S. Department of Education, 2023).

Moreover, assessing student–teacher correspondence and their degree of shared concern across school climate may support SBH teams in identifying areas of strength or concern. Larger discrepancies may indicate differences in awareness or sensitivity to student needs, which may hinder early identification of concerns and reduce the effectiveness of SBH supports. For example, substance abuse reports across students and teachers corresponded, yet students, on average, reported less concern related to substance abuse than teachers. Students may be experiencing or observing substance use-related issues differently than teachers; therefore, SBH teams may inquire about students’ awareness and access to substance use supports, teacher awareness of identification and referral processes, and assess whether Tier 1 prevention or Tier 2 targeted supports are adequately aligned to student needs.

Beyond safety-specific concerns, the broader school environment, including perceptions of physical support, opportunities for engagement, and discipline, plays important roles in shaping SBH implementation. Given that student and teacher perceptions significantly corresponded across the school environment, shared views regarding the general tone and function of the school’s contextual environment may align. For Mental Health, significant correspondence demonstrates that students and teachers similarly reported perceptions in the same direction; however, on average, students identified more concern related to mental health in their school than teachers, suggesting students and teachers may perceive their school’s mental health based on different experiences or interactions. For SBH teams, this may indicate further inquiry into the tiered mental health supports provided. At Tier 1, SBH teams may assess whether universal mental health promotion efforts, student-teacher relationships, and school-wide mental health literacy initiatives are visible, accessible, and responsive to students’ mental health needs, while also considering whether teachers have the training and mental health literacy needed to recognize and support student concerns. At Tier 2, SBH teams may assess whether identification and referral processes are clear and equitable, whether targeted supports are adequately addressing student needs, and whether both students and teachers are aware of available supports and how to access them (Gronholm et al., 2018; Thapa et al., 2013).

Although non-significant associations in engagement-related subdomains, such as school participation and instructional supports, had low correspondence in how students and teachers experience the school setting, these findings may still be informative. Engagement is closely tied to students’ willingness to participate in schoolwide and targeted supports (Taylor et al., 2017; Thapa et al., 2013). Low correspondence may suggest that students and teachers are drawing from different experiences when assessing these areas of school climate. For SBH, this may warrant additional inquiry before selecting interventions or allocating resources, as there may be differences in how students and teachers interpret engagement-related experiences. At Tier 1, low correspondence may suggest the need to strengthen student and/or teacher partnership, improve inclusive and culturally responsive schoolwide practices, or focus on relationship-building across students and staff (Barrett et al., 2015; Thapa et al., 2013). At Tier 2, low correspondence may suggest that students who are less connected to the school may also be less likely to participate in targeted supports (Zabek et al., 2023).

Taken together, these findings support the value of both student and teacher perspectives when assessing school climate. SBH teams may benefit from examining where perspectives correspond or diverge, which may guide responsive school improvement efforts. While safety concerns often demand immediate attention, broader environmental factors may shape the sustainability and depth of SBH efforts. Leveraging school climate data, particularly areas of correspondence between students and teachers, may enable schools to move beyond reactive responses and toward systemic, actionable improvements that create conditions conducive to sustained and equitable SBH implementation.

### 4.3. Limitations

Several limitations should be considered when interpreting these findings, including limitations that emerged from the larger study for which this study’s data is derived. First, the original study encountered complications regarding the school climate survey (SCS) administration and implementation across sites. The SCS varied in timing (each semester vs. varied semesters), delivery format (virtual vs. paper), and participation rates (whole school vs. individual classroom), leading to procedural inconsistencies and administration differences that may have introduced measurement error or contributed to site-level differences. Second, because the school climate data were collected cross-sectionally, causal inferences cannot be made. While significant correlations were found, longitudinal designs with standardized administration would improve data quality and enhance comparability across sites to draw causal conclusions. Third, the COVID-19 pandemic also impacted attrition rates for both student and teacher participants. Attrition may have led to findings that do not capture meaningful results of those who discontinued participation, impacting the study’s generalizability. Variability in teacher response rates across schools, including instances of very small teacher samples, may have also impacted the stability of the school-level data. Schools with minimal teacher participation may yield less reliable estimates of school climate perceptions, which should be considered when interpreting correlation results. Additional limitations of the original study, although not directly related to this study, are outlined in the forthcoming paper (see Weist et al., 2026 review).

While many data collection inconsistencies resulted from COVID-19-induced barriers, the most prominent data collection variabilities were identified between schools by state due to differences in state-level quarantine mandates. For example, schools in the mid-Atlantic state were required to stay home longer than schools in the Southern state, creating variability when schools were able to reimplement survey distribution and collection. Also, students and teachers completed the SCS during varying semesters across schools (Southern: spring 2020, spring 2021, fall 2021, spring 2022, fall 2022, and spring 2023; Mid-Atlantic: fall 2021, spring 2022, and spring 2023). Furthermore, unforeseen circumstances (such as school violence and other school-wide emergencies) also contributed to data inconsistencies across schools. As a result, the spring 2022 school climate dataset provided the most comprehensive data among the initially considered years. Longitudinal school climate research across multiple years of school participation would provide a more comprehensive understanding of school climate perspectives.

The exploratory analyses were conducted using aggregate school-level scores from 19 schools, which limited the statistical approaches available for examining correspondence. While Pearson correlations provided an appropriate exploratory measure of linear association between perceptions of school climate, they do not fully capture the correspondence between students and teachers. More advanced agreement-based approaches would require access to individual nested data and larger samples, which should be examined in the future. Another statistical limitation of this study was that we did not control for experiment-wise error across the multiple correlations examined to enable a more comprehensive exploration and identification of signals of correspondence and non-correspondence between students and teachers. Given the number of domains and subdomains analyzed, the probability of Type I error may be inflated. However, conservative corrections in this exploratory study would have reduced statistical power and increased the likelihood of overlooking potentially meaningful patterns. Therefore, findings should be interpreted as preliminary rather than confirmatory. Future research should consider applying corrections for multiple comparisons or using multivariate approaches to reduce the likelihood of spurious findings and to strengthen the robustness of the observed associations.

### 4.4. Future Directions

Future research should continue to examine both student and teacher perceptions of school climate for identifying key factors that may support the implementation of SBH programs. Moderate to strong correspondence between student and teacher perceptions of school safety and environment suggests some shared perceptions in these areas. Exploring how the correspondence *and* discrepancy of perspectives affect the identification of school needs and associated intervention response may support a comprehensive view of how school climate influences SBH implementation. Qualitative research may be instructive here. Specifically, improved understanding of how students and teachers view their school climate differently may help schools identify previously hidden barriers to academic achievement, emotional well-being, and behavioral health of students. Research tracking school climate perceptions over time may also identify whether improvements in corresponding perceptions of climate influence student, staff, and system outcomes. Such findings could give insight into whether correspondence between student and staff perceptions of climate could serve as an indicator of overall school well-being and capacity for sustaining effective SBH programming. Future research, including qualitative studies (e.g., focus groups with students and teachers), may also explore the mechanisms driving low correspondence, such as leadership support, communication, and external factors impacting the broader school community, to explore how these mechanisms may strengthen both school climate and SBH service delivery.

Further, identifying barriers to school climate may highlight areas of improvement in service delivery (Splett et al., 2023). Addressing these barriers may, in turn, improve workforce outcomes such as retention by reducing job stress and burnout (Luther et al., 2017). Intentionally leveraging school climate to improve school staff retention rates may serve as a strategic approach, as a stable workforce may improve continuity of care and promote higher implementation fidelity (Johnson-Kwochka et al., 2020). Relatedly, given that SBH depends on strong interdisciplinary collaboration (Splett et al., 2023), research should investigate how enhancing climate-informed professional development could influence staff retention and SBH program outcomes. Embedding consistent use of school climate data within decision-making processes may also enhance coherence across school initiatives and support a shared understanding of current school climate conditions. This data source may inform school team analyses, guiding responsive implementation improvements, deimplementation, and sustained successful SBH programs. Further research in these areas may provide critical insights for leveraging school climate to improve effective and sustainable behavioral health services in schools.

## 5. Conclusions

Consistent with prior research, our findings support that school climate plays a key role in shaping both student and teacher experiences within schools (Aldridge & McChesney, 2018; Franco et al., 2022; Hilts & Liu, 2023; Podiya et al., 2025). Shared perceptions of school climate may strengthen connection, trust and collaboration in sustaining the school’s overall well-being, conditions that are essential for effective SBH programs. Shared understanding in these domains may facilitate improved communication and stronger engagement with SBH programs across students and staff. As such, correspondence in school climate may enhance the school’s capacity to coordinate behavioral health supports, increase buy-in from family and community partners, and sustain implementation improvements for both student and staff outcomes.

Overall, this research emphasizes the importance of a healthy, collaborative school climate as a critical foundation for effective, sustainable SBH programming and implementation. Student and teacher perspectives may each provide unique insight into school strengths and needs, and may elucidate potential “blind spots” in how school climate concerns are identified and addressed. Schools aiming to improve behavioral health services may need to first address structural factors such as school climate and related conditions that impact student and staff outcomes before prioritizing intervention implementation. While the present study does not look to establish that correspondence directly improves SBH implementation, the findings support that comparing student and teacher perceptions for correspondence as well as discrepancies may offer a useful, data-informed strategy for identifying areas that warrant further inquiry. By prioritizing school-wide perceptions of school climate, schools can create environments that allow both students and staff to thrive, therefore sustaining effective and stable SBH implementation to support improved student outcomes.

## Figures and Tables

**Table 1:** Gender.

Characteristics		Students (N)	Teacher (N)
Gender	Male	614	263
	Female	493	36
	Non-binary/Gender nonconforming	40	1
	Other	29	3
	Prefer not to answer	12	0

**Table 2:** School climate participants by school.

School	Students (N)	Teachers (N)
1	72	16
2	72	49
3	126	24
4	71	10
5	50	18
6	66	35
7	62	36
8	26	30
9	71	1
10	49	6
11	68	6
12	34	10
13	62	4
14	13	11
15	69	11
16	44	1
17	49	9
18	119	3
19	65	19

**Table 3:** Descriptive statistics for student and teacher SCS.

	Students		Teachers		Cohen’s *d*
	*M*	*SD*	*M*	*SD*	*d*
Engagement Domain	290.80	14.49	257.95	20.84	1.86
Cultural & Linguistic Competence	282.76	19.18	256.59	19.05	1.37
Relationships	296.55	15.74	247.36	17.71	2.94
School Participation	287.03	15.58	264.62	30.99	0.96
Safety Domain	293.74	33.13	284.76	16.08	0.37
Emotional Safety	310.84	22.15	283.94	21.77	1.23
Physical Safety	297.49	38.93	283.88	28.49	0.40
Bullying/Cyberbullying	309.57	35.54	286.72	17.81	0.86
Substance Abuse	204.58	89.02	299.12	24.13	−1.67
Environment Domain	301.15	14.22	272.20	20.46	1.67
Physical Environment	322.63	31.30	277.48	39.93	1.27
Instructional Environment	281.55	12.54	288.34	13.50	−0.52
Physical Health	-	-	249.21	29.50	-
Mental Health	317.64	20.76	255.55	22.78	2.85
Discipline	284.43	13.09	276.69	24.98	0.41

School climate domains are highlighted in grey, with accompanied sub-domains below each.

**Table 4:** Student and teacher SCS correlations.

	Student ∗ Teacher Correlation	*p*-Value	Confidence Intervals (95%)
Engagement Domain	0.34	0.160	[−0.14, 0.69]
Cultural & Linguistic Competence	0.45	0.052	[−0.00, 0.75]
Relationships	0.28	0.238	[−0.19, 0.65]
School Participation	0.02	0.939	[−0.44, 0.47]
Safety Domain	0.75 ***	<0.001	[0.45, 0.90]
Emotional Safety	0.19	0.435	[−0.29, 0.59]
Physical Safety	0.58 **	0.009	[0.17, 0.82]
Bullying/Cyberbullying	0.69 **	0.001	[0.37, 0.87]
Substance Abuse	0.51 *	0.024	[0.08, 0.78]
Environment Domain	0.48 *	0.036	[0.04, 0.77]
Physical Environment	0.33	0.174	[−0.15, 0.68]
Instructional Environment	0.07	0.790	[−0.40, 0.50]
Mental Health	0.60 *	0.007	[0.20, 0.83]
Discipline	0.61 *	0.005	[0.22, 0.84]

* *p* < 0.05, ** *p* < 0.005, *** *p* < 0.0005. School climate domains are highlighted in grey, with accompanying sub-domains below each.

## Data Availability

The data presented in this study are available on request from the senior author (Dr. Weist), as analyses related to this study are still being conducted by the lead research team.
